# BReast CAncer susceptibility gene 2 deficiency exacerbates oxidized LDL‐induced DNA damage and endothelial apoptosis

**DOI:** 10.14814/phy2.14481

**Published:** 2020-07-07

**Authors:** Shweta Singh, Hien Nguyen, David Michels, Hannah Bazinet, Pratiek N. Matkar, Zongyi Liu, Lilian Esene, Mohamed Adam, Antoinette Bugyei‐Twum, Elizabeth Mebrahtu, Jameela Joseph, Mehroz Ehsan, Hao H. Chen, Mohammad Qadura, Krishna K. Singh

**Affiliations:** ^1^ Department of Medical Biophysics Schulich School of Medicine and Dentistry University of Western Ontario London ON Canada; ^2^ Anatomy and Cell Biology Schulich School of Medicine and Dentistry University of Western Ontario London ON Canada; ^3^ Division of Cardiology Keenan Research Centre for Biomedical Science and Li Ka Shing Knowledge Institute of St. Michael's Hospital Toronto ON Canada; ^4^ Institute of Medical Science University of Toronto Toronto ON Canada; ^5^ Department of Biology University of Western Ontario London ON Canada; ^6^ Vascular Surgery Keenan Research Centre for Biomedical Science and Li Ka Shing Knowledge Institute of St. Michael’s Hospital Toronto ON Canada; ^7^ Department of Surgery University of Toronto Toronto ON Canada; ^8^ Pharmacology and Toxicology University of Toronto Toronto ON Canada

**Keywords:** BRCA2, DNA damage, endothelial cell, endothelial dysfunction, oxidative stress

## Abstract

Mutations in the tumor suppressor gene BRCA2 (**BR**east **CA**ncer susceptibility gene **2**) predispose carriers to breast, ovarian, and other cancers. In response to DNA damage, BRCA2 participates in homology‐directed DNA damage repair to maintain genome stability. Genome‐wide association studies have identified an association between BRCA2 single nucleotide polymorphisms and plasma‐lipid levels and lipid deregulation in humans. To date, DNA damage, apoptosis, and lipid deregulation are recognized as central pathways for endothelial dysfunction and atherosclerosis; however, the role of BRCA2 in endothelial dysfunction remains to be elucidated. To determine the role of BRCA2 in endothelial dysfunction, *BRCA2* was silenced in human umbilical vein endothelial cells (ECs) and assessed for markers of DNA damage, apoptosis, and endothelial function following oxidized low‐density lipoprotein (oxLDL) treatment. OxLDL was found to induce significant reactive oxygen species (ROS) production in *BRCA2*‐silenced ECs. This increase in ROS production was associated with exacerbated DNA damage evidenced by increased expression and activation of DNA double‐stranded break (DSB) marker γH2AX and reduced RAD51‐foci formation—an essential regulator of DSB repair. Increased DSBs were associated with enhanced expression and activation of pro‐apoptotic p53 and significant apoptosis in oxLDL‐treated *BRCA2*‐silenced ECs. Loss of BRCA2 in ECs was further associated with oxLDL‐induced impaired tube‐forming potential and eNOS expression. Collectively, the data reveals, for the first time, a novel role of BRCA2 as a regulator of EC survival and function in the setting of oxLDL treatment *in vitro*. Additionally, the data provide important clues regarding the potential susceptibility of BRCA2 mutation carriers to endothelial dysfunction, atherosclerosis, and other cardiovascular diseases.

## Graphical abstract

We, for the first time, demonstrate that BRCA2 is basally expressed in endothelial cells. Loss of BRCA2 in endothelial cells significantly exacerbated oxLDL‐induced DNA damage and apoptosis indicating that BRCA2‐mutation positive individuals might be at increased risk of endothelial dysfunction–associated diseases.

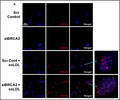

## 1 INTRODUCTION

Atherosclerosis, a chronic inflammatory disease and a leading cause of death worldwide, is characterized by endothelial dysfunction that plays an early and permissive role in the development and progression of the disease (Anderson et al., 1995; Paravicini & Touyz, 2006, 2008). The initial events in atherogenesis consist of subendothelial retention of low‐density lipoprotein (LDL) and its oxidative modification, which are followed by infiltration and activation of inflammatory cells (Pirillo, Norata, & Catapano, 2013). Oxidized LDLs (oxLDL) stimulate the expression of cell surface adhesion molecules of endothelial cells (ECs), which mediate the rolling and adhesion of blood leukocytes to the luminal wall of blood vessels; after adhesion to the endothelium, leukocytes migrate into the vessel intima under the effects of chemokines (Pirillo et al., 2013). Of these leukocytes, macrophages are activated as induced by oxLDLs which leads to the production and release of proinflammatory cytokines and reactive oxygen species (ROS), ultimately causing endothelial dysfunction and atherosclerotic plaque destabilization (Pirillo et al., 2013). Additionally, ROS can mediate the oxidative damage of DNA, including strand breaks and base and nucleotide modifications in sequences with high guanosine content (Bennett, 2001). Double‐stranded DNA breaks (DSBs) may activate the DNA damage response and DNA repair actors, including ATM (ataxia telangiectasia mutated), which proceed to phosphorylate and activate specific checkpoint kinases, such as CHK2, and subsequently, the tumor suppressor gene p53 (Bennett, 2001). In another study, p53 has been proposed as a regulator of macrophage proliferation in atherosclerosis (Martinet & Kockx, 2004). A common mechanism through which cardiovascular risk factors lead to endothelial dysfunction is increased oxidative stress, which in turn promotes DNA damage, EC dysfunction and apoptosis (Bennett, 2001).

Germline mutations in the tumor suppressor, *BRCA2* (**BR**east **CA**ncer susceptibility gene **2**), have commonly been associated with an increased risk of developing breast and ovarian cancers (Connor et al., 1997; Kinzler & Vogelstein, 1997; Yoshida & Miki, 2004). In fact, it is estimated that *BRCA2* mutants carry a 50%–60% lifetime risk of developing breast cancer. BRCA2 is classified as a “tumor suppressor” and “caretaker” based on its proposed roles in homology‐directed DNA damage repair (DDR) of DSBs, the cell cycle, and transcription (Connor et al., 1997; Kinzler & Vogelstein, 1997; Yoshida & Miki, 2004). Loss of BRCA2 results in defective repair, which when prolonged, can accumulate a lethal amount of damaged DNA causing cancer or apoptosis (Connor et al., 1997). DNA damage is a key factor not only in cancer, but also in diseases characterized by oxidative stress (Cai & Harrison, 2000; Herbst, Toborek, Kaiser, Mattson, & Hennig, 1999), a mechanism involved in endothelial dysfunction. Therefore, it is likely that BRCA2 plays an early role in cardiovascular diseases (CVDs) such as atherosclerosis (Gimbrone Jr. & Garcia‐Cardena, 2013; Herbst et al., 1999; Schachinger, Britten, & Zeiher, 2000; Shukla et al., 2011; Shukla, Singh, Yanagawa, Teoh, & Verma, 2010). There is increasing evidence for the accumulation of damaged DNA in atherosclerotic plaque, either as genomic alterations or as DNA adducts (Andreassi, Barale, Iozzo, & Picano, 2011; Izzotti, Cartiglia, Lewtas, & De Flora, 2001; Singh, Shukla, Quan, et al., 2013). BRCA1 and BRCA2 mutations have also been reported in CVDs, for example, mutations in BRCA1 and BRCA2 are shown to be associated with a two‐fold increased risk for diabetes (Bordeleau et al., 2011) and a 37% increase of all‐cause mortality risk for breast cancer patients mainly due to CVDs (Zhou, Zhang, Gu, & Xia, 2015). Excess non‐neoplastic deaths have been reported in individuals with BRCA1/2 mutations (Mai et al., 2009), and we have previously shown that BRCA1/2 play cardioprotective roles (Lovren et al., 2014; Shukla et al., 2011; Shukla et al., 2010;26; Singh, Shukla, Quan, et al., 2013; Singh et al., 2012; Singh, Shukla, Yanagawa, et al., 2013; Teoh et al., 2013). Moreover, a genetic analysis has identified a breast cancer‐related,* BRCA2* single nucleotide polymorphism associated with plasma lipid levels and CVD (Asselbergs et al., 2012; Miao et al., 2017), as well as lipid and metabolite deregulation in individuals with BRCA2 mutations (Ramadan et al., 2015; Stanwell & Gluch, 2015). DNA damage, lipid deregulation, and endothelial apoptosis and dysfunction are the central pathways of endothelial dysfunction and atherosclerosis (Hopkins, 2013; Singh & Jialal, 2006). However, the specific roles of BRCA2 in endothelial dysfunction and atherogenesis remain unknown. Our overall objective is to evaluate the role of endothelial BRCA2 in endothelial function and atherosclerosis using clinically relevant, *in vitro* methods.

Moreover, to gain insight about BRCA2 in an atherosclerosis animal model, we measured *BRCA2* in atherosclerotic plaques. Our qPCR data on aortic RNA isolated from ApoE^‐/‐^ mice fed a high‐fat diet for 8 weeks show significantly reduced *BRCA2* expression in comparison to the control ApoE^‐/‐^ mice fed a normal diet. Next, we confirmed basal BRCA2 expression in ECs and also demonstrated that oxLDL significantly down‐regulated BRCA2 expression in ECs. We then, for the very first time, demonstrated that in *BRCA2*‐silenced ECs, oxLDL significantly increased ROS production, DNA damage, apoptosis, and reduced DNA damage repair and endothelial function (tube‐forming potential); indicating a significant role for BRCA2 in ECs.

## 2 MATERIALS AND METHODS

### 2.1 Cell culture, *BRCA2*‐silencing and oxLDL

Human Umbilical Vein ECs (HUVECs, pooled, Lonza) were cultured in EC growth medium‐2 (EGM™‐2 Bulletkit™; Lonza) containing growth factors or MCDB 131 (Gibco) supplemented with serum and antibiotics. HUVECs from passage number 4–6 were used in the experiments. siRNA‐mediated *BRCA2* gene knockdown was conducted with 5 nM siBRCA2 or scrambled control (Ambion) and the DharmaFECT‐4 transfection reagent (Dharmacon) according to the manufacturer's guidelines. Cells were serum‐starved overnight and then treated with different doses (0, 5, 10, 20, 50, and 100 μg/ml) of oxLDL. Human LDL (Alfa Aesar) was purchased and oxidized using 20 μM CuSo_4_ in PBS at 37°C for 24 hr.

### 2.2 Quantitative real‐time PCR

The Quantitect kit (Qiagen) was utilized to synthesize complementary DNA (cDNA), which were then subjected to quantitative polymerase chain reaction (qPCR) using the ABI ViiA 7 Real‐Time PCR System (Applied Biosystems). For PCR, SYBR^®^ Select Master Mix (Applied Biosystems) were mixed with forward and reverse primers for human *BRCA2* (forward – 5’gccccttcacttcagcaat‐3’ and reverse 5’‐acaaatagacgaaaggggca‐3’), *p21* and human *GAPDH*, as well as mouse *BRCA2* and mouse *GAPDH* in accordance with the manufacturer's instructions as previously described (Murugavel et al., 2018; Singh, Shukla, Quan, et al., 2013).

### 2.3 Immunoblot, immunofluorescence and flow cytometry

HUVECs were harvested 24, 48, and 72 hr posttransfection with either siBRCA2 or scrambled control. Cell lysates were prepared in RIPA buffer (Sigma) to isolate total protein. Protein was loaded on sodium dodecyl sulfate (SDS) polyacrylamide gels for immunoblotting analysis for (1:1000 dilution) BRCA2 (Cell Signaling # 9012), γH2AX (Cell Signaling # 2577), H2AX (Cell Signaling # 2595), Cleaved caspase‐3 (Cell Signaling # 9661), ATM (Abcam # ab78), p(phospho)ATM (Abcam # 36810), pCHK1 (Cell Signaling # 2341), CHK1 (Cell Signaling # 2360), p53 (Cell Signaling # 2524), Bax (Cell Signaling # 2772), Bcl‐2 (Abcam # ab59348), p21 (Abcam # ab7960), peNOS (Millipore # 07‐428), eNOS (BD Biosciences # 610296), RAD51 (Santa Cruz # sc‐8319), and GAPDH (Millipore # MAB374). Immunoblots were developed using an enhanced chemiluminescence substrate (SuperSignal^TM^, Life Technologies) and a superior ChemiDoc^TM^ imaging system (Bio‐Rad). Densitometry was performed to measure the band intensities using the ImageJ software. Immunofluorescence was performed in 4‐chamber microscopy slides as previously described (Matkar et al., 2016; Murugavel et al., 2018; Singh et al., 2015, 2020), and immunofluorescence signals from γH2AX (Cell Signaling) and RAD51 (Cell Signaling) staining were visualized with standard protocols 24 hr post‐treatment. Fluorescent microscopy images were captured using the Zeiss LSM700 confocal microscope and processed using ZEN imaging software. Apoptosis was further confirmed *via* quantifying HUVECs positive for annexin V‐fluorescein isothiocyanate (FITC) and propidium iodide (PI) with flow cytometry.

### 2.4 Proliferation, *in vitro* angiogenesis and ROS measurements

HUVECs were seeded at a density of 1–1.5 × 10^4^ cells/well in 96‐well plates, transfected with siBRCA2 or scrambled control and then treated with oxLDL, and cell proliferation was evaluated using the WST‐8 Cell Proliferation Assay Kit (Cayman Chemicals) according to the manufacturer's instructions. The angiogenic properties of HUVECs were examined using the In Vitro Angiogenesis Assay Kit (Chemicon): HUVECs were seeded onto ECMatrix Gel–coated 96‐well plates (Millipore, Billerica, Mass) at a density of 7–9 × 10^3^/well and the extent of their angiogenesis was determined with the aid of a phase‐contrast microscope (Nikon). Each experiment was performed thrice in triplicates. HUVECs underwent silencing before they were treated for 24 hr with oxLDL. ROS were determined using the OxiSelect ROS Assay Kit (Cell Biolabs, Inc).

### 2.5 Animal studies

All animal‐related experiments and procedures were approved by the St Michael's Hospital Animal Care Committee and performed in accordance with the guidelines of the Canadian Council on Animal Care. Male ApoE^−/−^ mice were obtained from The Jackson Laboratory (Bar Harbor, Me). A group of 8‐week‐old ApoE^−/−^ mice were fed the Western diet for 8 weeks. Control ApoE^−/−^ mice were fed a normal diet. These mice were subsequently euthanized, and their aortas were isolated for RNA extraction.

### 2.6 Statistical analysis

Data were analyzed *via* Student's *t*‐test, to compare the means of two groups. Differences between multiple means of more than two groups were calculated by analysis of variance and in the case of overall differences, individual means were compared posthoc with the Bonferroni's test. Data are presented as the mean ± *SD* unless otherwise indicated. A *p* < .05 was considered to indicate statistical significance.

## 3 RESULTS

### 3.1 Reduced expression of BRCA2 in the aorta of the atherosclerosis animal model and oxLDL‐treated ECs

**FIGURE 1:**
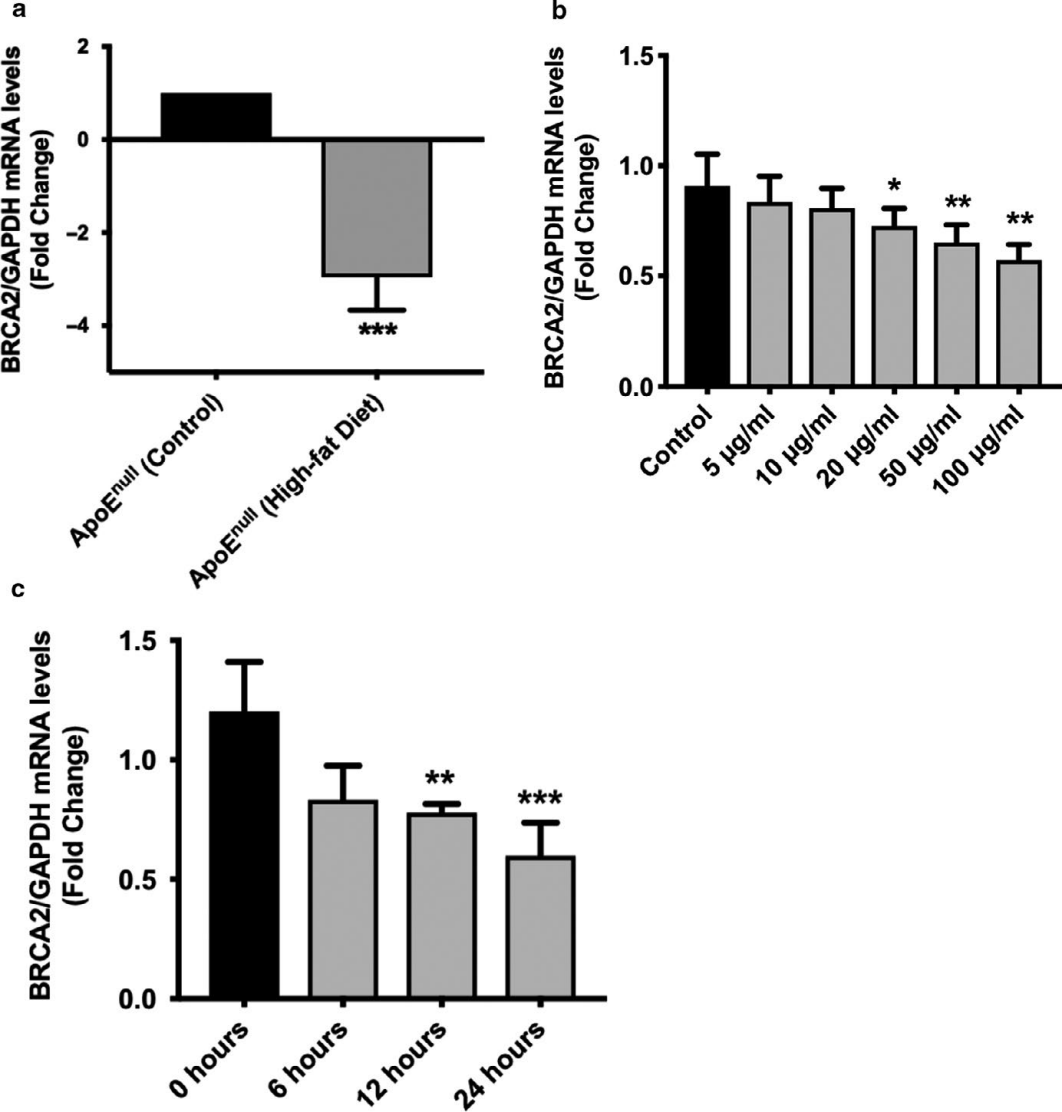
Reduced expression of *BRCA2* in the aorta of the atherosclerosis animal model and in oxLDL‐treated HUVECs. (a) *BRCA2* qPCR data on RNA isolated from aorta of ApoE^−/−^ mice fed high‐fat diet *versus* control diet. *N* = 6/group. ***p < .001 *versus* control. (b) *BRCA2* qPCR performed on RNA isolated from oxLDL (0, 5, 10, 20, 50, and 100 μg/ml for 24 hr)‐treated HUVECs. *N* = 3–4 in triplicate. *,**p < .05, .01 *versus* control. (c) *BRCA2* qPCR performed on RNA isolated from oxLDL (100 μg/ml for 0, 6, 12 and 24 hr)‐treated HUVECs. *N* = 3–4 in triplicate. **,***p < .01, .001 *versus* control

To investigate the potential involvement of BRCA2 in the atherogenic process in an atherosclerosis animal model, we evaluated the expression level of *BRCA2* in the aorta of the high‐fat diet fed ApoE^‐/‐^ (ApoE^null^) mice and compared it with the expression of *BRCA2* in normal diet fed ApoE^‐/‐^ mice. As demonstrated, we observed a significantly reduced *BRCA2* expression level in the high‐fat diet fed ApoE^‐/‐^ mice in comparison to the normal diet fed ApoE^‐/‐^ mice (Figure 1a). In order to get an insight about the cause and/or effect relationship of BRCA2 with atherosclerosis in ECs, we next measured *BRCA2* expression levels in ECs treated with different doses (0, 5, 10, 20, 50, and 100 μg/ml) of oxLDL for 24 hr and measured *BRCA1* and *BRCA2* expression. We observed a significant reduction in the *BRCA2* expression level in response to oxLDL in a dose‐dependent manner, but *BRCA1* expression remained unaffected (Figure 1b; Figure S1a). We then treated ECs with 100 μg/ml of oxLDL and evaluated BRCA1 and BRCA2 expression 0, 6, 12, and 24 hr posttreatment. We observed a significant time‐dependent downregulation only for *BRCA2* with maximum downregulation at 24 hr, whereas *BRCA1* remained unaffected for all the studied time‐points (Figure 1c; Figure S1b). These findings prompted us to investigate the role of endothelial BRCA2 at baseline and after oxLDL treatment to ECs.

### 3.2 Oxidized LDL‐induced ROS production, DNA damage, and apoptosis is exacerbated in BRCA2‐deficient endothelial cells

**FIGURE 2:**
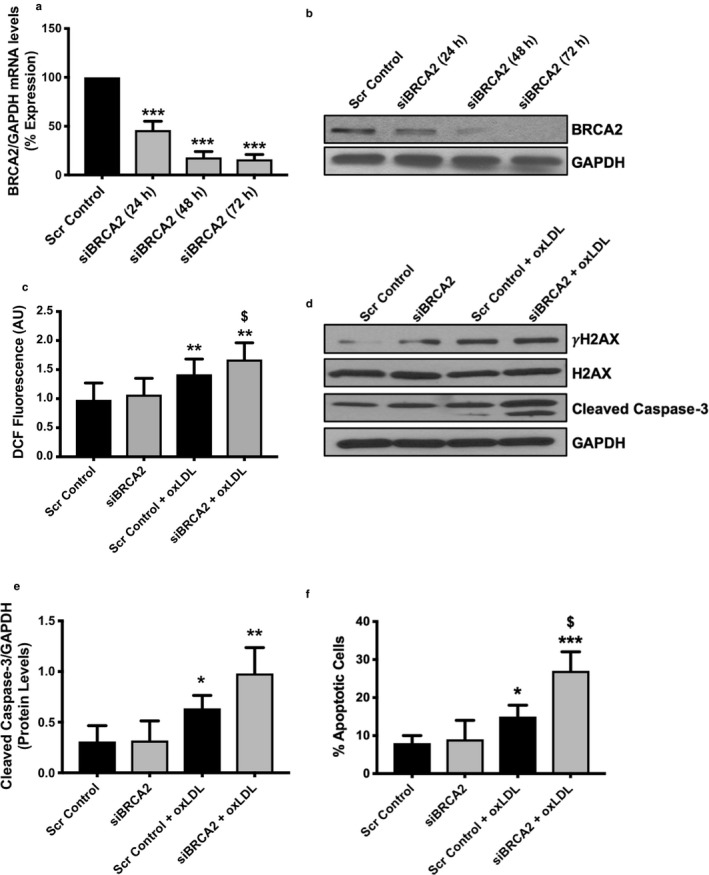
OxLDL‐induced ROS, DNA damage, and apoptosis is exacerbated in BRCA2‐deficient endothelial cells. HUVECs were transfected either with scrambled control or siBRCA2, and RNA and protein were extracted 24, 48, and 72 hr post‐transfection to perform (a) qPCR and (b) immunoblotting for BRCA2. (c) ROS was measured in HUVECs after transfecting with either scrambled control or siBRCA2 for 48 hr and then treated with oxLDL (100 μg/ml) for 12 hr. Twelve‐wells/group. (d) Immunoblotting for γH2AX, H2AX, and cleaved caspase‐3 in HUVECs transfected with either scrambled control or siBRCA2 for 48 hr and then treated with oxLDL (100 μg/ml) for 24 hr. (e) quantification for the protein levels of cleaved caspase‐3. GAPDH was used as a loading control. (f) Apoptosis was also measured by flow cytometry in HUVECs transfected with either scrambled control or siBRCA2 for 48 hr and then treated with oxLDL (100 μg/ml) for 24 hr. *N* = 3–4 in triplicates. *, ** and ***p < .05, 0.01 and 0.001 *versus* scrambled control, ^$^ *p* < .05 *versus* scrambled control + oxLDL

**FIGURE 3:**
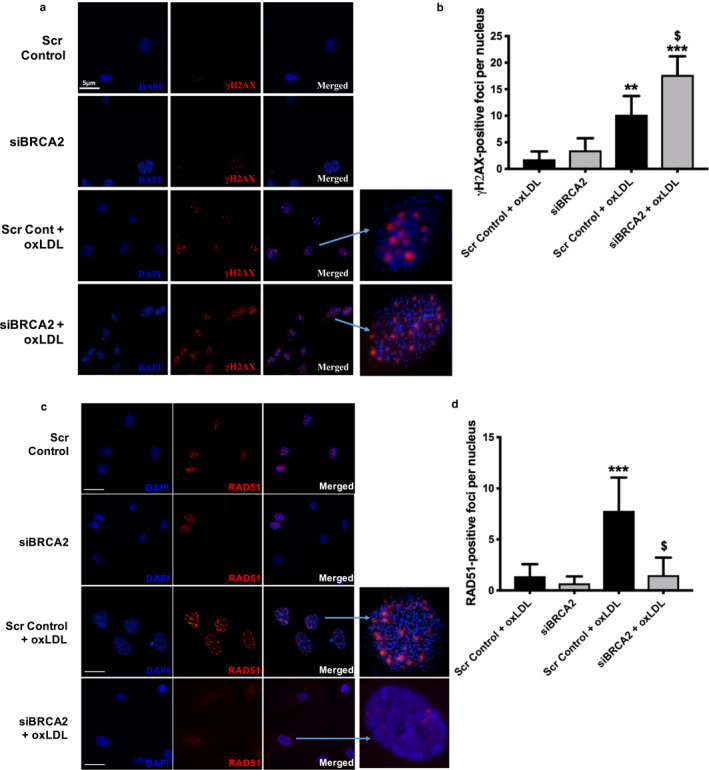
Endothelial cell‐specific loss of BRCA2 exacerbates oxLDL‐induced DNA damage and impairs DNA damage repair. Immunofluorescence and quantification for the DNA damage marker (a and b) γH2AX (red fluorescent) and DNA damage repair (c and d) RAD51 (red fluorescent) in HUVECs transfected with either scrambled control or siBRCA2 for 48 hr and then treated with oxLDL (100 μg/ml) for 24 hr. ** and ***p < .01 and 0.001 *versus* scrambled control, ^$^ *p* < .05 *versus* scrambled control + oxLDL. Enlarged γH2AX‐ and RAD51‐ (red fluorescent) positive nuclei (blue; DAPI) to clearly visualize the foci formation in HUVECs. The blue arrow indicates the respective enlarged nuclei. Bar for scrambled control and siBRCA2 is 5 μm, scrambled control + oxLDL is 2.5 μm and siBRCA2 + oxLDL is 3.5 μm in figure (d)

To evaluate the functional relevance of BRCA2 in ECs, we utilized loss‐of‐function approaches. The optimal dose of siBRCA2 was determined to be 5 nM. siBRCA2–mediated loss of BRCA2 was confirmed by qPCR and immunoblotting 24, 48, and 72 hr post‐transfection in siBRCA2‐transfected ECs in comparison to the scrambled control‐transfected ECs (Figure 2a,b). All subsequent studies were performed in three groups of HUVECs; non‐transfected, siBRCA2‐transfected, and scrambled control‐transfected HUVECs. Findings from the non‐transfected and scrambled control‐transfected HUVECs were indistinguishable; therefore, the data from non‐transfected HUVECs are not presented. Oxidized LDL induces the generation of ROS, which is a critical mediator of EC dysfunction and apoptosis (Valente, Irimpen, Siebenlist, & Chandrasekar, 2014; Xu et al., 2008), and also induces DSBs (Sallmyr, Fan, & Rassool, 2008). In order to understand the effect of oxLDL on ROS, we measured the ROS generation in the scrambled control and siBRCA2‐transfected ECs. Expectedly, we observed significantly enhanced ROS production in the oxLDL treated ECs, which was further exacerbated in the BRCA2‐silenced ECs in comparison to the control cells (Figure 2c). Next, since ROS‐associated oxidative stress induces double‐stranded breaks (DSBs) and BRCA2 repairs DSBs, we evaluated the effect of oxLDL on the generation of DSBs in the BRCA2‐deficient ECs. With this aim, we measured the extent of DSBs by measuring the expression level of γH2AX *via* immunoblotting as the γH2AX foci formation is the first cellular response to DSBs (Caresana, Tenti, & Di Matteo, 1990). Our immunoblotting data on γH2AX demonstrated that oxLDL was able to induce significant DNA damage, which was further exacerbated in BRCA2‐deficient ECs (Figure 2d). ROS induces DNA damage that ultimately leads to apoptosis (Kocyigit & Guler, 2017), and EC apoptosis plays an important role in endothelial dysfunction and the atherogenic pathway (Valente et al., 2014). We then treated siBRCA2 and scrambled control‐transfected HUVECs with oxLDL for 24 hr and measured apoptosis. OxLDL triggered significant apoptosis in the siBRCA2‐transfected HUVECs in comparison to the scrambled control‐transfected HUVECs (Figure 2d–f). The immunoblotting data on γH2AX were further confirmed by immunofluorescence, which also demonstrated more extensive γH2AX staining indicating exaggerated DNA damage in oxLDL‐treated BRCA2‐deficient ECs when compared with that seen in the scrambled control‐transfected ECs (Figure 3a,b). BRCA2 physically interacts with the DNA damage repair molecule RAD51; following DNA damage, BRCA2 translocates RAD51 into the nucleus. This BRCA2/RAD51 focus formation is a critical and essential step for the repair of damaged DNA (Yuan et al., 1999). To evaluate the effect of the loss of BRCA2 on DNA damage repair, we measured the extent of RAD51‐foci formation in oxLDL‐treated scrambled control and siBRCA2‐transfected ECs. Our immunofluorescence data clearly demonstrated significantly reduced RAD51‐foci formation in the oxLDL‐treated, BRCA2‐deficient ECs in comparison to the control ECs indicating a defective repair that results in the accumulation of damaged DNA in BRCA2‐deficient ECs (Figure 3c,d).

### 3.3 Oxidized LDL induces increased expression of DNA damage repair‐associated molecules, p53, and p53‐related apoptotic molecules in BRCA2‐deficient ECs

**FIGURE 4:**
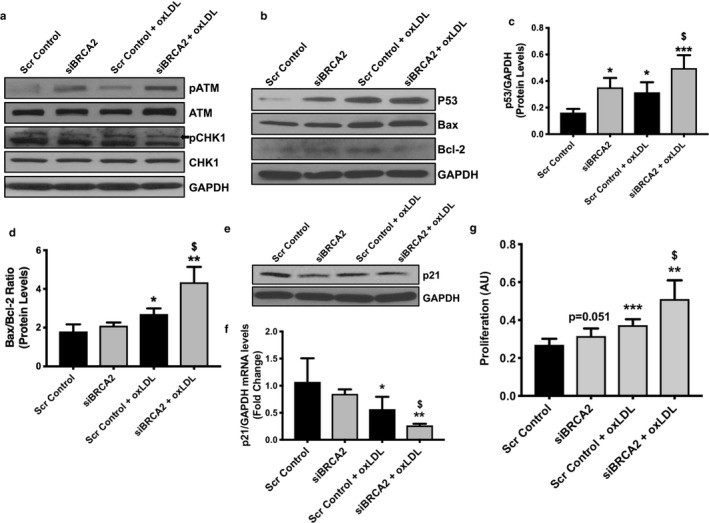
Oxidized LDL induces increased expression of DNA damage‐related molecules, p53, and related apoptotic molecules in BRCA2‐deficient endothelial cells. Proteins were extracted from HUVECs transfected either with scrambled control or siBRCA2 for 48 hr and then treated with oxLDL (100 μg/ml) for 24 hr to perform immunoblot for (a) p(phospho)ATM, ATM, pCHK1, and CHK1, (b) p53, Bax, and Bcl‐2, (e) p21. GAPDH was used as a loading control. Protein quantification for p53 (c) and for the Bax/Bcl‐2 ratio (d). (f) qPCR for *p21*. (g) Proliferation was evaluated in HUVECs transfected either with scrambled control or siBRCA2 for 48 hr and then treated with oxLDL for additional 24 hr. *N* = 3–4/group for immunoblot and qPCR in triplicates. Six‐wells/group for proliferation. *, ** and ***p < .05, 0.01 and 0.001 *versus* scrambled control, ^$^ *p* < .05 *versus* scrambled control + oxLDL, *p* = .051 *versus* scrambled control

Under the crisis of DNA damage, eukaryotic mitotic cells either induce apoptosis or activate the checkpoint pathways in order to arrest the cell cycle and commence DNA repairs. DSB sensors such as ATM and checkpoint kinase CHK1/2 function as DNA damage response effectors as they phosphorylate p53, which in turn can promote cell cycle arrest through cyclin‐dependent kinase inhibitor 1 (p21) for DNA repair, and may induce apoptosis if the damage is irreparable (Hoeijmakers, 2001). Immunoblots for total and activated ATM and CHK1 demonstrated increased activation of these proteins isolated from siBRCA2‐transfected, oxLDL‐treated ECs in comparison to scrambled control‐transfected, oxLDL‐treated ECs (Figure 4a). p53 upregulation is sufficient to induce apoptosis, as the loss of p53 partly rescued the embryonic lethality observed in systemic BRCA2 knockout mice (Ludwig, Chapman, Papaioannou, & Efstratiadis, 1997). Therefore, we proceeded to determine whether the observed increased apoptosis and DSBs in oxLDL‐treated, BRCA2‐deficient ECs are associated with the upregulation of p53. Accordingly, we observed significantly increased expression of p53 at both transcript and protein levels in the oxLDL‐treated, BRCA2‐deficient ECs (Figure 4b,c). In the p53‐dependent survival and/or apoptotic signaling pathway, the shift in the ratio of the level of pro‐apoptotic Bax to that of the pro‐survival molecule Bcl‐2, both of which are fundamental regulators of cellular apoptosis and/or survival, is a critical factor that determines whether the cells would undergo one of two fates (Basu & Haldar, 1998). The observed oxLDL‐mediated increased apoptosis in BRCA2‐deficient ECs was associated with a decrease in pro‐survival Bcl‐2 levels and an increase in pro‐apoptotic Bax protein, yielding a ∼4‐fold increase in the Bax to Bcl‐2 ratio, thus shifting the balance within the cells from survival to an apoptotic mode (Figure 4b,d). To evaluate the effect of oxLDL‐induced increased p53 and DNA damage in BRCA2‐deficient EC proliferation, next, we measured the expression level of cyclin‐dependent kinase inhibitor 1 (p21) and cell proliferation. The oxLDL treatment significantly downregulated p21 expression at both the transcript as well as protein levels, which was further downregulated in the oxLDL‐treated, BRCA2‐deficient ECs (Figure 4e,f). BRCA2 deficiency appears to increase the rate of EC proliferation, but these changes were not significant for three of the studied time points (24, 48, and 72 hr; Figure S1c). However, oxLDL significantly induced EC proliferation, which was further exacerbated in BRCA2‐deficient ECs. (Figure 4g). Overall, our data demonstrate that the loss of BRCA2 exacerbated oxLDL‐induced ROS production, DNA damage, apoptosis, and induced proliferation in ECs (Figures 3 and 4).

### 3.4 Endothelial cell‐specific loss of BRCA2 diminishes endothelial eNOS production and tube‐forming potential of ECs

**FIGURE 5:**
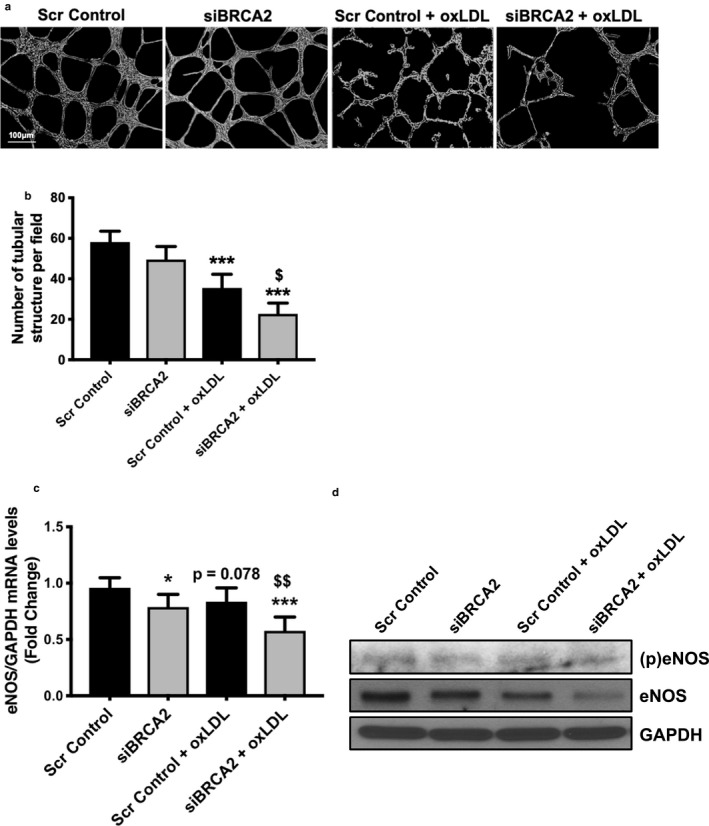
Endothelial cell‐specific loss of BRCA2 exacerbates oxLDL‐induced endothelial dysfunction. (a) Representative micrographs and (b) quantification showing capillary‐like tube formation 5 hr post‐oxLDL (100 μg/ml) treatment to scrambled control‐ or siBRCA2‐ transfected HUVECs for 48 hr. Six‐wells/group. ***p < .001 *versus* scrambled control, ^$^ *p* < .05 versus scrambled control + oxLDL. (c) qPCR for eNOS and (d) immunoblotting for (p)eNOS, eNOS, and GAPDH in HUVECs transfected with either scrambled control or siBRCA2 for 48 hr and then treated with oxLDL (100 μg/ml) for 24 hr. *, ***p < .05, 0.001 versus scrambled control, ^$$^ *p* < .01 versus scrambled control + oxLDL, *p* = .078 versus scrambled control

Angiogenesis plays an important role in the process of atherogenesis (Herrmann, Lerman, Mukhopadhyay, Napoli, & Lerman, 2006). One of the most widely used *in vitro* assays to model angiogenesis and endothelial function is the tube formation assay. OxLDL significantly impaired the capacity of ECs to form capillary‐like tubular structures; such an effect was observed to deteriorate in BRCA2‐deficient ECs (Figure 5a,b). Endothelial nitric oxide synthase (eNOS) plays fundamental roles in the maintenance of endothelial function and angiogenesis, as disruptions in eNOS expression or activation can culminate in endothelial dysfunction (Kolluru, Siamwala, & Chatterjee, 2010). We measured the expression and activation level of eNOS in oxLDL‐treated BRCA2‐deficient endothelial cells and, to our surprise, oxLDL and loss of BRCA2 both led to a decrease in the transcript and protein expression and activation level of eNOS, potentially exacerbating the impaired tube formation in the oxLDL‐treated, BRCA2 deficient ECs in comparison to the control ECs (Figure 5c,d). Such findings suggest an essential role of BRCA2 in endothelial function.

## 4 DISCUSSION

Atherosclerosis, a leading cause of morbidity, is a chronic inflammatory condition characterized by EC apoptosis and endothelial dysfunction, which play a major role in the progression and in the clinical course of the disease (Cai & Harrison, 2000; Gimbrone Jr. & Garcia‐Cardena, 2016). In many cases, a common mechanism through which cardiovascular risk factors culminate in endothelial dysfunction and atherogenesis is *via* increased oxidative stress, which in turn induces DNA damage, as well as EC dysfunction and apoptosis (Bordeleau et al., 2011; Izzotti et al., 2001). Excessive production of ROS, a family of free radical molecules that includes molecular oxygen and its derivatives produced in all aerobic cells and the associated outstripping of endogenous antioxidant defense mechanisms, have been implicated in typical processes in which ROS oxidize biological macromolecules, such as DNA, protein, carbohydrates, and lipids such as LDL—a major step in the development of atherosclerosis (Zhou et al., 2015). oxLDL is a known common culprit behind the generation of oxidative stress in the vascular system and subsequent endothelial dysfunction (Singh, Shukla, Yanagawa, et al., 2013). In addition, DNA damage and repair mechanisms as responses are activated in oxLDL‐mediated, injured ECs (Mai et al., 2009).

Increasing evidence has emerged indicating the occurrences and accumulation of DNA damage in atherosclerotic plaque as either genomic alterations or DNA adducts (Andreassi et al., 2011; Izzotti et al., 2001; Singh, Shukla, Quan, et al., 2013). As BRCA2 is canonically known to respond to DNA damage by participating in the DDR pathway, a previous study demonstrated that both BRCA1/2 mutation carriers are potentially at higher cardiovascular risk (Arts‐de Jong, Maas, Massuger, Hoogerbrugge, & de Hullu, 2014). Our previous work on BRCA1 also showed a protective role of BRCA1 in endothelial dysfunction and atherosclerosis along with other CVDs (Lovren et al., 2014; Shukla et al., 2011; Singh, Shukla, Quan, et al., 2013; Singh, Shukla, Yanagawa, et al., 2013; Teoh et al., 2013). Moreover, lipid and metabolite deregulation is observed in humans carrying either BRCA1 or BRCA2 (BRCA1/2) mutations and a genetic analysis identified BRCA2 SNPs’ association with plasma‐lipid levels (Ramadan et al., 2015; Stanwell & Gluch, 2015), which plays an important role in endothelial dysfunction and atherosclerosis (Asselbergs et al., 2012). BRCA2 SNP rs9534275 not only is associated with LDL and total cholesterol but also with increased risk of coronary artery disease and ischemic stroke (Miao et al., 2017) along with increased risk for benign breast cancer (NCBI: ClinVar). These, along with other evidence, indicate a causative role of the BRCA1/2 genes in CVDs (Bordeleau et al., 2011; Lovren et al., 2014; Mai et al., 2009; Shukla et al., 2011; Shukla et al., 2010; Singh, Shukla, Quan, et al., 2013; Singh et al., 2012; Singh, Shukla, Yanagawa, et al., 2013; Teoh et al., 2013; Zhou et al., 2015). Research investigating the biological functions of the proteins encoded by BRCA1/2 have dominated the field since they were identified with speculated critical roles in the analysis of families at high risk from breast and ovarian cancer. BRCA1/2 encode large and nuclear‐localized proteins that are widely expressed in different tissues during the S and G2 phases, have little resemblance to one another or to proteins of known function and, albeit they function within a common pathway of DNA damage repair, possess distinct functional domains that interact with different proteins in different stages of the process (Fridlich, Annamalai, Roy, Bernheim, & Powell, 2015; Roy, Chun, & Powell, 2012; Venkitaraman, 2001). Between BRCA1 and BRCA2, a key difference lies in the cancer predisposition rate, with BRCA1 mutants carry a higher lifetime risk (70%–80%) of developing breast cancer than BRCA2 (50%–60%; Roy et al., 2012). Lifetime risk, estimated frequency, types (such as ovarian, prostate, pancreatic and others) and characteristics (such as estrogen‐receptor expression) of cancers also vary between individuals with BRCA1 and BRCA2 mutations (Roy et al., 2012). However, only mutations in BRCA2, and not in BRCA1, have been reported in sporadic cancers (Roy et al., 2012). In addition, while BRCA1 appears omnipresent at a functional level, with a multitude of biochemical data describing multiprotein interactions, BRCA2 appears to exert more of a direct role to promote DDR (Davies et al., 2001; Yang et al., 2002). Therefore, the differential role played by BRCA1 and BRCA2 warrants independent studies to elucidate their respective roles in endothelial biology.

In the present study, in order to understand the role of BRCA2 in endothelial dysfunction and apoptosis in atherosclerosis, we measured the expression level of BRCA2 in the aorta of the high‐fat diet *versus* normal diet fed ApoE^‐/‐^ mice. We observed significantly reduced BRCA2 expression in the high‐fat diet fed ApoE^−/−^ mice in comparison to the normal diet fed ApoE^−/−^ mice (Figure 1a). Next, to understand the relationship between atherosclerosis, the endothelium, and BRCA2 expression, we measured BRCA2 expression levels in oxLDL‐treated ECs and observed a significantly reduced BRCA2 expression following oxLDL treatment in a dose‐ and time‐dependent manner (Figure 1b,c). It is interesting to note that, although the expression level of BRCA1 in endothelial cells was similar to BRCA2, the BRCA1 expression was not significantly affected following all used doses of oxLDL treatment and for all the studied time‐points in endothelial cells (Figure S1a,b).

As a next step, we proceeded to silence the *BRCA2* gene to assess its significance in the setting of endothelial dysfunction (Figure 2a,b). Oxidative modification and subendothelial retention of LDL represent the initial event in atherosclerosis. In fact, oxidized LDL activates ECs, which leads to the release of ROS and proteolytic enzymes involved in matrix degradation and ultimately atherosclerotic plaque destabilization (Lovren et al., 2014) Thus, in our experiments, we used oxLDL as a stressor to induce ROS generation, DNA damage, and apoptosis. Furthermore, following the loss of BRCA2, we consistently observed a trend of increasing ROS, DNA damage, and apoptosis with the addition of oxLDL treatment to the ECs (Figures 2 and 3). Consistently, oxLDL treatment increased γH2AX protein expression and immunofluorescence, suggesting an increasing presence of DNA double‐stranded breaks, which further increased in the *BRCA2*‐silenced ECs (Figures 2d and 3a,b). Measurements of cleaved‐caspase 3 protein expression revealed a similar trend—apoptosis was magnified with the loss of BRCA2 in ECs (Figure 2d,e). We confirmed apoptosis by flow cytometry, which also confirmed exacerbated oxLDL‐induced apoptosis in BRCA2‐deficient ECs in comparison to oxLDL‐treated, control ECs (Figure 2f). For the first time, we have shown that loss of BRCA2 exacerbates ROS generation, DNA damage, and apoptosis (Figures 1 and 3). It is likely that BRCA2 works to protect against DNA damage caused by oxLDL and promotes DNA repair pathways. Indeed, researchers have already demonstrated BRCA2's primary role in DNA damage repair (Marmorstein, Ouchi, & Aaronson, 1998). It is interesting to note that oxLDL‐induced ROS was further exacerbated in BRCA2‐silenced ECs (Figure 2c). BRCA1 is known to possess pro‐survival, anti‐oxidant activity (Gorrini et al., 2013) and provide resistance against oxidative stress (Bae et al., 2004), but there are no studies evaluating the anti‐oxidant activity of BRCA2. Our ROS data indicates that BRCA2 appears to play an anti‐oxidant role against oxLDL and warrants investigation in this direction. Overall, our data show that BRCA2 is required to protect against oxLDL‐induced ROS, DNA damage, and apoptosis to preserve endothelial function.

Next, we measured several sensors of DNA damage and modulators of apoptosis. Following DNA damage, dividing cells either undergo cell arrest by activating checkpoint pathways to repair the DNA damage or, if irreparable, these cells undergo p53‐mediated apoptosis (Greenberg, 2008; Hoeijmakers, 2001). ATM and checkpoint kinase CHK1/2, generally DSB sensors, promote p53‐mediated apoptosis or p21‐mediated cell cycle arrest (Greenberg, 2008). Although the rule governing DNA damage and apoptosis is unclear, p53 provides an important link (Basu & Haldar, 1998). p53 interacts with BRCA2 and regulates DDR, growth arrest, and apoptosis (Hakem, de la Pompa, & Mak, 1998; Marmorstein et al., 1998). The importance of the p53‐BRCA2 axis is well illustrated in studies where embryonic lethality of BRCA2 knockout mice was rescued by the elimination of one *p53* allele (Hakem et al., 1998). At the transcriptional level, using human vascular smooth muscle cells, it was also found that several genes related to DNA damage and repair were up‐regulated in the presence of oxLDL (Damian‐Zamacona et al., 2016). Moreover, oxLDL causes apoptosis *via* increased p53 levels, such that induction of p53 alone is sufficient to kill cells (Basu & Haldar, 1998; Cheng, Cui, Chen, & Du, 2007). Interestingly, p53 co‐localizes with apoptotic cells in atherosclerotic lesions (Cheng et al., 2007; Tabas, 2001). Our results point toward an important role of the BRCA2/p53 signaling axis in CVDs. Therefore, we hypothesized that EC‐specific loss of BRCA2 would exacerbate oxLDL‐induced DNA damage sensor activity and p53 function, leading to increased apoptosis and endothelial dysfunction *in vitro*. Accordingly, we observed increased activation of DNA damage sensors; ATM and CHK1, which was further associated with an increased expression of p53 and a shift towards a p53‐associated pro‐apoptotic Bax/Bcl2 ratio (Figure 4a–c). Our data show that in BRCA2‐deficient ECs, oxidative stress results in increased levels of p53, leading to increased apoptosis and thereby setting the stage for endothelial apoptosis and dysfunction.

To further evaluate the effect of oxLDL‐induced increased p53 and DNA damage in BRCA2‐deficient ECs, we measured the expression level of p21 and cell proliferation. Oxidized LDL induced significant downregulation of p21, which was further reduced in oxLDL‐treated BRCA2‐deficient ECs in comparison to oxLDL‐treated control ECs (Figure 4e,f). Loss of BRCA2 in ECs also appeared to down‐regulate p21 at both protein and transcript levels; however, these changes were non‐significant (Figure 4e,f). Given the down‐regulation of cell cycle inhibitor p21 in oxLDL‐treated BRCA2‐deficient and control ECs, we expected an increased proliferation rate following oxLDL treatment in both BRCA2‐deficient and control ECs. Accordingly, we observed a significant increase in the EC proliferation rate in the oxLDL‐treated control group, which was further increased in oxLDL‐treated, *BRCA2*‐silenced ECs (Figure 4g). To our surprise, we also observed a strong trend toward increased rates of proliferation in BRCA2‐deficient ECs in comparison to control EC, but these changes were not significant (Figure S1c). We repeated this experiment several times and observed a similar trend. This may be attributed to the sensitivity of the method used or to the efficiency of *BRCA2* silencing.

We then evaluated the effect of BRCA2 loss, especially in the setting of oxLDL treatment, on the angiogenic potential of the ECs and did observe significant differences in capillary‐like tube formation in ECs. Cells treated with oxLDL had a markedly reduced amount of tubular structures; however, this was further reduced with both oxLDL treatment and loss of BRCA2 (Figure 5a,b). Endothelial function regulator eNOS plays a central role in regulating endothelial function and angiogenesis (Heiss, Rodriguez‐Mateos, & Kelm, 2015). To grasp an insight about the mechanism associated with the reduced potential of tube formation in oxLDL‐treated BRCA2‐deficient ECs, we measured the expression and activation level of eNOS at the transcript and protein level (Kolluru et al., 2010). Interestingly, our data demonstrated reduced expression as well as a reduced activation level of eNOS, suggesting its role in the loss of BRCA2 and oxLDL‐associated, reduced tube formation in ECs (Figure 5c,d). Notably, there was a clear reduction in eNOS expression at the transcript (*p* = .078) and protein level in the oxLDL‐treated control cells and BRCA2‐silenced cells, and both appeared to have an additive effect in the BRCA2‐silenced, oxLDL‐treated ECs, causing impaired tube‐forming potential (Figure 5).

These data warranted further investigation and prompted us to evaluate our *in vitro* findings in a more detailed atherosclerosis animal model. Accordingly, we are in the process of generating endothelial cell‐specific BRCA2 knockout mice with an ApoE^−/−^ background and then evaluating atherosclerosis in order to delineate the cause and effect relationship between endothelial BRCA2 and atherosclerosis. The mechanism behind oxLDL‐induced BRCA2, as well as p21 and eNOS downregulation is important but still remains to be elucidated. This is clinically relevant as heterozygous mutations in BRCA2 lead to haploinsufficiency in BRCA2 mutation‐carrying, breast and ovarian cancer patients. Oxidized LDL‐induced oxidative stress in these BRCA2 mutation–positive individuals would further downregulate the expression of BRCA2, causing a situation with an almost complete loss of endothelial BRCA2, which might further exacerbate endothelial dysfunction, apoptosis, and atherosclerosis, warranting further investigation in humans. Altogether, our data elucidate a novel role of BRCA2 in EC survival, apoptosis, and endothelial function. These data indicate potential susceptibility of BRCA2 mutation carriers to endothelial dysfunction and other related cardiovascular complications such as atherosclerosis. Furthermore, our data indicate that therapeutic strategies aiming to upregulate endothelial BRCA2 might be protective, particularly in the setting of vascular diseases characterized by oxidative stress such as atherosclerosis.

## DISCLOSURE

None.

## AUTHOR CONTRIBUTION

KKS conceived and designed the study. HN, DM, HB, PNM, ZL, LE, MA, ABT, ME, and HHC and KKS carried out the experiments and analyzed the data. SS, HN, JJ, EM, MQ, and KKS helped improve the discussion. SS and KKS wrote and assembled the manuscript with final figures.

## Supporting information



Figure S1
