# EXPRESSION OF CONCERN: BReast CAncer susceptibility gene 2 deficiency exacerbates oxidized LDL‐induced DNA damage and endothelial apoptosis

**DOI:** 10.14814/phy2.71121

**Published:** 2026-10-06

**EXPRESSION OF CONCERN**: S. Singh , H. Nguyen , D. Michels , H. Bazinet , P.N. Matkar , Z. Liu , L. Esene , M. Adam , A. Bugyei‐Twum , E. Mebrahtu , J. Joseph , M. Ehsan , H.H. Chen , M. Qadura , and K.K. Singh , “BReast CAncer susceptibility gene 2 deficiency exacerbates oxidized LDL‐induced DNA damage and endothelial apoptosis,” Physiological Reports 8, no. 13 (2020): e14481, 10.14814/phy2.14481.PMC734084532638521

This Expression of Concern is for the above article, published online on 7 July 2020 in Wiley Online Library (wileyonlinelibrary.com), and has been issued by agreement between the journal Editor‐in‐Chief, Josephine C. Adams; the American Physiological Society; The Physiological Society; and Wiley Periodicals, LLC. Concerns were raised by a third party, as well as on PubPeer [1], regarding highly similar micrographs in Figure 5A of this article and Figure 1A of a different article by some of the same authors (Murugavel et al. 2018: https://doi.org/10.3389/fphar.2018.00737), for different experimental conditions. Due to a laboratory move several years ago, the authors were unable to provide the original data. The editor notes that the potentially incorrect image panel in Figure 5A does not alter the scientific conclusions of the article. Additionally, the authors have subsequently published on endothelial‐specific BRCA2 knockout mice, showing that endothelial BRCA2 deficiency promotes atherosclerosis in vivo (https://doi.org/10.48550/arXiv.2306.10993). However, in the absence of the original data, the journal and publisher are unable to fully investigate the discrepancies. Therefore, the journal has decided to issue an Expression of Concern to inform and alert readers. The authors have been informed about this Expression of Concern.
