# Supplementary material for: BReast CAncer susceptibility gene 2 deficiency exacerbates oxidized LDL‐induced DNA damage and endothelial apoptosis
Source: Physiol Rep. 2020 Jul 7;8(13):e14481. doi: 10.14814/phy2.14481 (PMC7340845; doi:10.14814/phy2.14481)
Supplement: Supplementary file 1 — Figure S1 [file PHY2-8-e14481-s001.pdf]

SUPPLEMENTARY FIGURE 1

A

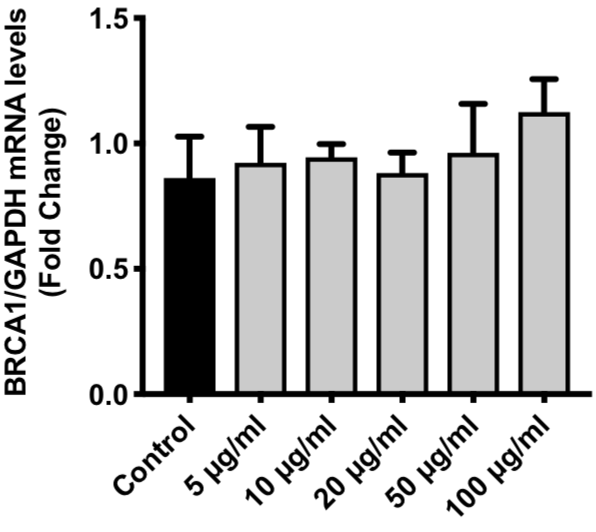

B

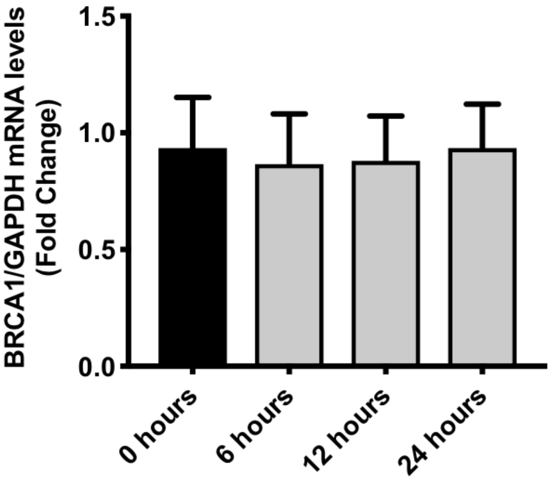

C

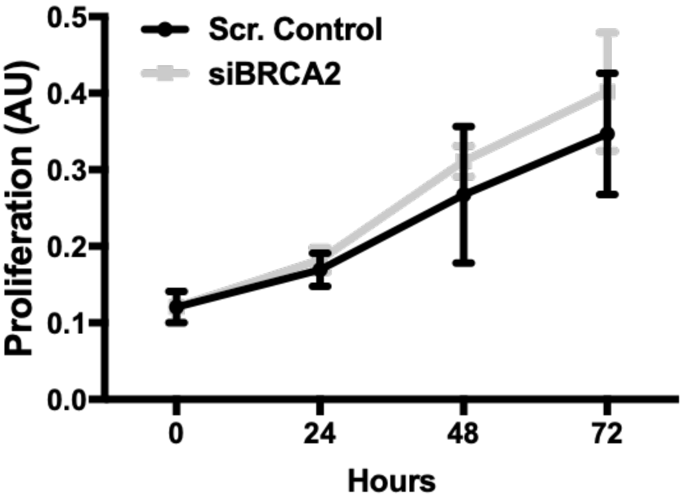

Supplementary Figure 1. (A) BRCA1 qPCR performed on RNA isolated from oxLDL (0, 5, 10, 20, 50 and 100 µg/mL for 24 hours)-treated HUVECs. N = 3-4 in triplicate. **(B)** BRCA1 qPCR performed on RNA isolated from oxLDL (100 µg/mL for 0, 6, 12 and 24 hours)-treated HUVECs. N = 3-4 in triplicate. **(C)** Proliferation was evaluated in HUVECs transfected either with scrambled control or siBRCA2 for 24, 48 and 72 hours. N = 12.
